# Annual Address before the New York Medico-Chirurgical Society

**Published:** 1883-05

**Authors:** 


Annual Address before the New York Medico-Chirurgical Society. By the President, E. P. Fowler, M. D. 1882.

This address consists in a very pedantic rehash of the old-time, oft-refuted, allopathic criticisms of Hahnemann and Homœopathy. It is therefore not worthy of comment.

The learned President has evidently not heard of Hahnemann's labors in drug proving, etc., for he says: "The extent to which drugs act as curatives, and the conditions to which their employment is adapted, furnishes a subject worthy of a serious attention which it has not yet received."

Hahnemann and Hering having failed to do any effective work in therapeutics, it is to be hoped that Dr. Fowler will allow his gigantic intellect to give it some "serious attention."

